# Standard reference values of weight and maximum pressure distribution in healthy adults aged 18–65 years in Germany

**DOI:** 10.1186/s40101-020-00246-6

**Published:** 2020-11-30

**Authors:** D. Ohlendorf, K. Kerth, W. Osiander, F. Holzgreve, L. Fraeulin, H. Ackermann, D. A. Groneberg

**Affiliations:** 1 Institute for Occupational Medicine, Social Medicine and Environmental Medicine, Goethe-University Frankfurt, Frankfurt am Main, Germany; 2 School of Dentistry, Department of Orthodontics, Goethe-University Frankfurt, Frankfurt am Main, Germany; 3 Institute of Biostatistics and Mathematical Modeling, Goethe-University Frankfurt, Frankfurt am Main, Germany

**Keywords:** Standard reference values, Healthy adults, Pressure distribution, Postural control, Pressure measuring plate

## Abstract

**Background:**

The aim of this study was to collect standard reference values of the weight and the maximum pressure distribution in healthy adults aged 18–65 years and to investigate the influence of constitutional parameters on it.

**Methods:**

A total of 416 healthy subjects (208 male / 208 female) aged between 18 and 65 years (Ø 38.3 ± 14.1 years) participated in this study, conducted 2015–2019 in Heidelberg. The age-specific evaluation is based on 4 age groups (G1, 18–30 years; G2, 31–40 years; G3, 41–50 years; G4, 51–65 years). A pressure measuring plate FDM-S (Zebris/Isny/Germany) was used to collect body weight distribution and maximum pressure distribution of the right and left foot and left and right forefoot/rearfoot, respectively.

**Results:**

Body weight distribution of the left (50.07%) and right (50.12%) foot was balanced. There was higher load on the rearfoot (left 54.14%; right 55.09%) than on the forefoot (left 45.49%; right 44.26%). The pressure in the rearfoot was higher than in the forefoot (rearfoot left 9.60 N/cm^2^, rearfoot right 9.51 N/cm^2^/forefoot left 8.23 N/cm^2^, forefoot right 8.59 N/cm^2^). With increasing age, the load in the left foot shifted from the rearfoot to the forefoot as well as the maximum pressure (*p* ≤ 0.02 and 0.03; poor effect size). With increasing BMI, the body weight shifted to the left and right rearfoot (*p* ≤ 0.001, poor effect size). As BMI increased, so did the maximum pressure in all areas (*p* ≤ 0.001 and 0.03, weak to moderate effect size). There were significant differences in weight and maximum pressure distribution in the forefoot and rearfoot in the different age groups, especially between younger (18–40 years) and older (41–65 years) subjects.

**Discussion:**

Healthy individuals aged from 18 to 65 years were found to have a balanced weight distribution in an aspect ratio, with a 20% greater load of the rearfoot. Age and BMI were found to be influencing factors of the weight and maximum pressure distribution, especially between younger and elder subjects. The collected standard reference values allow comparisons with other studies and can serve as a guideline in clinical practice and scientific studies.

## Introduction

### Weight and pressure distribution and its relevance for postural control

In the postural system, the foot has to fulfill a variety of functions—it is an organ of touch, support, and movement. It ensures that the joints above it are balanced under static and dynamic conditions in order to guarantee a secure stand. A biomechanical characteristic of the foot is that it is exposed to constant alternating loads [1]. Complex interactions of the sensory, motor, vestibular, and visual systems guarantee the maintenance of the balance [2] of movements or center of gravity displacements, which also cranially trigger the balance shift causing the body weight to be distributed to both lower extremities in a manner that does not cause the person to fall [3]. In a bipedal stand, the body weight is evenly distributed over both feet, each 50% of the load [1, 4]. If the shift in the center of gravity changes minimally, this relation is changed so that one foot has to carry more total load. Therefore, pressure plates are a suitable instrument for measuring balance and postural stability [5–11]. When the body weight is transferred, the pressure is distributed most strongly in the hindfoot, then decreasingly in the midfoot and forefoot. The distribution corresponds to 60% (rearfoot) to 40% (midfoot and forefoot) [1]. According to Obens [12], the rearfoot is loaded with 66% and the forefoot with 33%. Similar values were found by Scharnweber et al. [13] for men between 18 and 35 years with a load ratio between the back and forefoot (63.00%/36.67%). This was confirmed in a study by Ohlendorf et al. [14] who demonstrated a load distribution between the left and right foot of 50.1%:49.9% in female adults aged between 21 and 30 years (25 ± 2.7 years) in Germany. An additional load on the forefoot is also discussed by Lalande et al. [15]. Ohlendorf et al. [14] also found a lower load on the forefoot compared to the rearfoot (33.3%:66.7%), where the main load was in the right rearfoot (34.3%). These results are in agreement with those of Cuccia [16], which also demonstrated a higher load in the rearfoot (left 9.60 N/cm^2^, right 9.51 N/cm^2^) than in the forefoot (left 8.23 N/cm^2^, right 8.59 N/cm^2^) [17]. Lalande et al. [15] also report a higher load on the rearfoot compared to the forefoot. Ohlendorf et al. [14] determined that the balance distribution between the left and right foot is 49.91%:50.09% in young German women. This means that the rearfoot is loaded more than the forefoot (66.67%:33.3%). The greatest weight is placed on the right rearfoot (34.34%). There is a proven correlation between body weight, plantar pressure, and foot pain [18, 19]. Increasing body weight increases plantar pressure. Plantar pressure peaks are associated with foot pain [20]. An increase in body weight increases the intensity of pain [21]. The overall proprioception of the foot is determined not only by pressure transmission but also by the ankle, tendons, and proprioception of the long foot muscles [22]. They collect sensomotoric information to maintain overall balance. The long foot muscles, with their tension in the longitudinal direction, ensure that the arch is prevented from sinking against the spreading force. In addition, they ensure that the foot can twist with its partial joints in such a way that it adapts itself favorably with its bearing surface and can balance the multi-unit column above it by means of the ankle joints [23]. Cuccia [16] has determined an even pressure load between the left and right foot (621.35 g/cm^2^/626.67 g/cm^2^). Additionally, the main pressure load is centrally located under the forefoot [24–26]. Both when standing and walking, the maximum pressure values are higher at the III metatarsal head than under the metatarsal head I and V. Maetzler et al. [27] determine an increased pressure value under the II and III metatarsal bones, the big toe, and the heel. These results are also comparable with Bryant et al. [26], Hughes et al. [28], and Putti et al. [29]. A weakening of the connective tissue, as occurs in the case of age atrophy of the plantar fat pad or due to previous diseases such as rheumatism, leads to the loss of the natural buffering properties of the forefoot. This causes local pressure peaks and can lead to metatarsalgia [30].

### Influence of BMI on posture control

In overweight persons, the load on the medial longitudinal arch is approximately three times greater than in a normal-weight person [31]. This can cause negative biodynamic changes and possibly limit quality of life and physical activity [31]. Obese adults have more anomalies in the longitudinal medial arch, plantar fascia, increased plantar pressure, and balance problems compared to normal-weight adults [32]. The association between obesity, posture, fear of falling, and risk of falling is demonstrated in the study by Neri et al. [33], where obesity is associated with a risk of falling due to reduced postural balance and increased fear of falling. In their measurements of postural stability in overweight and obese men, Rezaeipour [34] concludes that weight gain is associated with disturbances of balance. According to the “Study of Adult Health in Germany” (DESG1) [35], 67.1% of men and 53.0% of women in the 18–79 age group are overweight. With increasing socio-economic status, the proportion of obese men decreases. This is consistent with the results of the study by Mensink et al. [36]. Overweight influences body stability [37, 38]. Hue et al. [39] also demonstrated that increasing weight correlates with a shift of the body’s center of gravity to the frontal, which in turn has a direct effect on postural control and foot load. It was observed that increasing BMI leads to increased variation in frontal and sagittal plane. Ohlendorf et al. [17] could not detect any significant changes in truck drivers in terms of foot pressure load sorted by BMI groups. Among the static parameters, body mass index was found to have a positive correlation with total plantar force (*r* = 0.50, *p* = 0.000) and total contact area (*r* = 0.33, *p* = 0.019). Only middle foot peak pressure (*r* = 0.32, *p* = 0.025) among the dynamic pedobarographic parameters had positive correlation with body mass index.

### Influence of age and gender on posture control

Pomarino et al. [40] and Lalande et al. [15] did not found an influence of gender in load distribution when considering adults. It is different for children: in growth, girls show significant advantages due to a developmental advantage over boys in postural control. In the elderly, Wolfson et al. [41] found increased postural instability in women (Ø 76 years), which was particularly pronounced when there was a reduction in somatosensory and visual input. This leads to a greater frequency of falls. The increase in instability with increasing age is often proved, too [42–46]. Schwesig et al. [45] describe the greatest postural stability at the age of 20.1–30 years. After the age of 50, there is a decrease in performance [47]. Mittermaier and Fialka-Moser [44] also come to this conclusion, but describe that performance increases again at the age of 60.1–70. Changes in postural control with increasing age also have been found by Røgind et al. [48].

Therefore, the aim of this study was to establish standard values of weight and maximum pressure distribution at the age of 18–65 years. Standard values can indicate changes in body weight and maximum pressure distribution before treatment and validate changes associated with any treatment or can classify, e.g., the severity of postural control deviations.

The following hypotheses were as follows:

1. Representative standard values for the weight and the maximum pressure distribution of healthy men and women between 18 and 65 years of age can be determined with the associated mean and median values, tolerance ranges (upper/lower limit), and confidence interval (left/right limit).
2. An increased BMI leads to an increased pressure load in the foot.
3. Age has no influence on the balance distribution.
4. Gender has no influence on the distribution of stress.

## Methods

### Subjects

A total of 416 (208 male/208 female) participants aged 18 to 65 years, with an average age of 38.3 (± 14.1) years, volunteered in this study. The body weight range was between 49.09 and 155.94 kg (Ø 72.86 ± 16.9 kg) and the height between 1.54 and 2.00 m (Ø 1.73 ± 0.09 m). This resulted in body mass indices of 14.89–45.56 kg/m^2^, yielding an average of Ø 24.24 ± 4.56 kg/m^2^. From these results, the following subdivisions using the WHO definition [49] were made: 6.79% of the participants were underweight (BMI < 18.5 kg/m^2^), 54.62% of the participants were normal weight (BMI 18.5–24.9 kg/m^2^), 29.08% were overweight/pre-obese (BMI 25–29.99 kg/m^2^), 7.61% were obese class I (BMI 30 - 34.99 kg/m^2^), 1.09 % obese class 2 (BMI 35.0– 39.99 kg/m^2^), and 0.81% were obese class 3 (BMI ≥ 40 kg/m^2^). In addition, the following age groups were formed: group 1, 18–30 years (77 m/85f); group 2, 31–40 years (47 m/34f); group 3, 41–50 years (38 m/31f); and group 4, 51–65 years (48 m/56f).

As inclusion criteria in this study, all subjects felt healthy according to subjective assessment. Chronic diseases, diseases of nervous system, or pregnant women are not allowed to be part in this study. Subjects with reported (head, ankle, spine, hip, knee) injuries, joint replacements, accidents involving these areas, or any sort of bodily injury that could influence how a person stood as well as ongoing orthodontic or orthopedic treatment were excluded from this study, too. This was determined using a questionnaire and led to the exclusion from the study. Written informed consent was obtained from all subjects.

The study was in accordance with the 1964 Helsinki Declaration and its later amendments and was approved by the local medical ethics committee of the Faculty of Medical Science, Goethe University Frankfurt, Germany (No. 219 / 14).

### Measurement system

The pressure measuring platform FDM-S (Zebris medical GmbH/Isny) was used. This consists of a 69 × 40 cm and 2.1 cm high measuring plate with a 54 × 33 cm sensor area (measuring area). In total, 46 × 64 capacitive force sensors are located on the plate, which are arranged in a matrix and work with a measuring accuracy of ± 5% (FS) and a delay rate of ≤ 3% (FS). The measuring range is between 1 and 120 N/cm^2^. The sensors are scanned at a measuring frequency of 120 Hz.

The evaluation software ABW-Med V3 determines weight distribution (%) and maximum pressure (N/cm^2^) (Table 1). The used range is determined over all measurements, i.e., the largest/smallest X or Y position with a pressure value not equal to 0 is searched for. This range is divided into quadrants in the middle between the minimum/maximum X or Y positions (for the left and right foot, as well as the left and right forefoot and rearfoot). The value specified by the software is the average of all time steps.

**Table 1:** Description of the evaluated weight distribution, force, and pressure parameters as well as the covered area, ellipse, and COP-length parameters. Confidence ellipse refers to the mathematical compensation ellipse of the COP trace. The confidence interval is assumed to be 2ϭ (Heidelberg, 2015)

Weight distribution	
Balance (%)	Weight distribution between right and left foot. The mean value over the entire measuring sequence is given.
Balance forefoot (%)	Weight distribution of forefoot separated for left and right foot. The mean value is given over the entire measuring sequence.
Balance rearfoot (%)	Weight distribution of the back foot for the left and right foot. The mean value over the entire measuring sequence is given.
**Pressure parameter**	
Maximum pressure (*N*/cm^2^)	Maximum pressure, a maximum value of the entire measuring sequence is specified.
Maximum pressure per foot (N/cm^2^)	Maximum pressure for left and right foot separated. The maximum value of the entire measuring sequence is indicated.
Maximum pressure forefoot (N/cm^2^)	Maximum pressure in forefoot, separated for left and right foot. The maximum value of the entire measuring sequence is indicated.
Maximum pressure rearfoot (N/cm^2^)	Maximum pressure in the rearfoot, separated for the left and right foot. The maximum value of the entire measuring sequence is indicated.

### Examination procedure

Each participant was instructed to stand in a habitual body position on the plate. The participants were urged to place themselves barefoot on the plate without external influences (e.g., shoes, stockings). Arms should hang down loosely with the view fixed at a point on the opposite wall on eye level. In addition, subjects were instructed not to move during the measurements. The foot position was taken habitually by each test person, but a spacer bar behind the feet ensures that both feet are completely captured in the measuring area of the plate and are at the same height. The standing width within the platform area and the rotation of the foot were not specified.

Each measurement lasted 5 s, the measurement being carried out a total of five times. An average of these five measurements was determined and used for further analysis. Since this measurement is part of a study in which the postural control is recorded simultaneously with a three-dimensional back scan, the measurement duration of a measurement sequence of 5 s had to be taken, since recording the upper body posture with the back scanner takes just that time. Prior to the study, several familiarization measurements were carried out to do justice to the shortened measurement duration.

### Statistical evaluation

The data were analyzed using the statistic program BiAS 11.0 (Epsilon Verlag, Darmstadt, Germany). The data were first tested for normal distribution by the Kolmogorov-Smirnov-Lilliefors test. According to the (not) existing of normal distribution appropriate tests were used for calculating mean, median, two-sided 95% confidence interval (CI), and tolerance range (TR)

TR was determined which describes the upper and lower limit within which 95% of all standard values of the test persons were located. The tolerance region (syn. “reference region” or “normal region”) was calculated according to Proschan [50] and Fraser [51] depending on an underlying normal distribution as a parametric or nonparametric region. A tolerance region describes the upper and lower limit of a region covering 95% of all standard values of the reference persons in question [50–52].

The Friedman test including a post hoc test was used for comparisons between the age groups. The data were then subjected to a Bonferroni-Holm correction.

The comparison of the two gender was performed using the Wilcoxon-Mann-Whitney *U* test.

Correlations between the metric parameters were examined by simple, linear correlation according to Pearson (parametric) or by rank correlation according to Spearman and Kendall (non-parametric) and were determined. For the effect size, the correlation coefficient rho was used according to different classes [53–55]. The effect size classification is as follows: (1 = < 0.2, poor; 2 = 0.2–0.4, weak; 3 = 0.4–0.6, moderate; 4 = 0.6–0.8, strong; 5 = > 0.8, optimal). The significance level was set at 5%.

## Results

### Tolerance range and confidence interval

All mean and median values, their tolerance range, and confidence interval are shown in Table 2.

**Table 2:** Mean value, tolerance range (lower and upper limit), and confidence interval (left and right limit) of all evaluation parameters. Non-normally distributed values are in italics (Heidelberg, 2015)

	Mean value/Median	Tolerance range Lower limit	Tolerence range Upper limit	Confidence interval Left limit	Confidence interval Right limit
Weight distribution (%)					
Balance left	50.07	27.72	77.82	49.12	51.03
Balance right	50.12	22.24	72.89	49.14	51.09
Forefoot left	*45.49*	19.78	*81.07*	*42.81*	*46.60*
Forefoot right	*44.26*	17.40	*78.21*	*43.61*	*47.05*
Rearfoot left	54.14	22.89	82.75	52.64	55.64
Rearfoot right	*55.09*	*21.75*	*82.50*	*53.37*	*56.94*
Pressure parameter (N/cm^2^)					
Maximum pressure	*12.5*	*7.00*	*21.35*	*12.00*	*13.00*
Left	*11.05*	*5.25*	*19.19*	*10.50*	*11.60*
Right	*11.00*	*6.34*	*19.38*	*10.60*	*11.50*
Forefoot left	8.23	3.00	16.81	7.83	8.59
Forefoot right	8.59	3.54	16.50	8.22	8.96
Rearfoot left	*9.60*	*3.54*	*18.20*	*9.00*	*10.00*
Rearfoot right	9.51	3.50	17.76	9.12	9.89

The body weight distribution on the left and right feet averaged 50.07% and 50.12%, respectively (TR lower limit, 27.72%/77.82%; upper limit, 22.24%/72.89%; CI left border, 49.12% and 49.14% (left) and 51.03% and 51.09% (right)). There was a balanced weight relationship between both feet, resulting in a balanced posture. On average, there was less load on the forefoot (left 45.49%, right 44.26%) and more load on the rearfoot of the left and right (54.14%, 55.09%). The maximum pressure of both feet was 12.5 N/cm^2^ (TR lower/upper limit, 7.00 N/cm^2^/21.35 N/cm^2^; CI left/right limit, 12.0 N/cm^2^ / 13.0 N/cm^2^). The median of the maximum pressure on the left (11.05 N/cm^2^) equaled the median of the maximum pressure on the right (11.0 N/cm^2^), indicating a uniform pressure load between the left and right foot. The median values of the left and right forefoot (8.23 N/cm^2^ and 8.59 N/cm^2^) were very similar. The median value of the left rearfoot was 9.60 N/cm^2^ while the mean of the right rearfoot was 9.51 N/cm^2^, demonstrating a higher pressure load on the left and right rearfoot compared to the forefoot left / right.

### Correlation between weight distribution/maximum pressure and age/BMI

Table 3 summarizes the correlations of the weight distribution and maximum pressure with age and BMI.

**Table 3:** Significant values of the correlation of age, height, and BMI. Balance parameters and COP-length: *p* value and Rho-value. The effect size classification is as follows: 1 = < 0.2, poor; 2 = 0.2–0.4, weak; 3 = 0.4–0.6, moderate; 4 = 0.6–0.8, strong; 5 = > 0.8, optimal. Non-normally distributed values in italics. Significant *p* values are highlighted in bold (Heidelberg, 2015)

	*p* value	Rho-value	*p* value	Rho-value
	Age		BMI	
Weight distribution (%)				
Balance left	0.99	0.01^1^	0.39	0.04^1^
Balance right	1.00	0.01^1^	0.38	− 0.04^1^
Forefoot left	**0.01**	0.13^1^	***0.001***	− *0.16*^1^
Forefoot right	*0.30*	*0.05*^1^	***0.001***	− *0.19*^1^
Rearfoot left	**0.02**	− 0.11^1^	**0.001**	0.17^1^
Rearfoot right	*0.48*	− *0.03*^1^	***0.001***	*0.17*^1^
Pressure parameter (N/cm^2^)				
Maximum pressure	*0.81*	*0.01*^1^	***0.001***	*0.31*^*2*^
Left	*1.00*	*0.001*^1^	***0.001***	*0.30*^*2*^
Right	*0.61*	*0.03*^1^	***0.001***	*0.29*^*2*^
Forefoot left	**0.01**	0.20^2^	***0.03***	*0.11*^1^
Forefoot right	**0.02**	0.12^1^	***0.001***	*0.35*^*2*^
Rearfoot left	***0.03***	− *0.11*^1^	***0.001***	*0.41*^*3*^
Rearfoot right	0.71	− 0.02^1^	**0.001**	0.41^3^

**Fig. 1:**
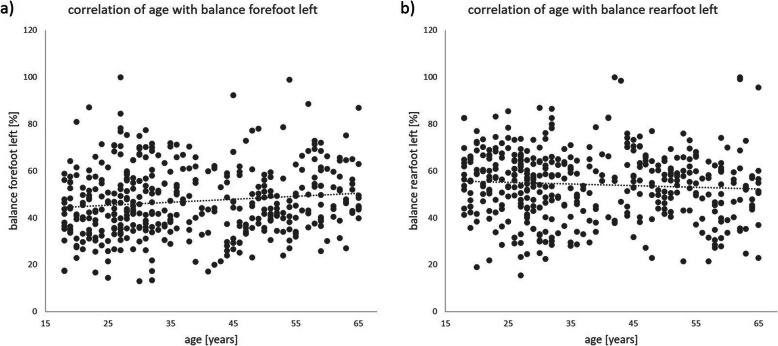
Correlation of age with **a** balance forefoot left and **b** balance rearfoot left

Increasing age was found to have an influence on the balance of the left forefoot (*p* ≤ 0.01, effect size poor) and the left foot (*p* ≤ 0.02, effect size poor) (Fig. 1). The pressure on the left and right forefoot reached significant values (*p* ≤ 0.01 and *p* ≤ 0.02, effect size poor and weak). At the rearfoot left, the *p* value was *p* ≤ 0.03 with the effect size poor. Analogous to the balance of the left rearfoot, the pressure in the rearfoot decreased to the left with increasing age (Fig. 1).

The BMI was found to correlate with the body weight distribution (Table 3); increasing BMI led to more balance in the forefoot on the left (*p* ≤ 0.001, effect size poor)/right (*p* ≤ 0.001, effect size 1 poor), whereas the balance in the rearfoot on the left (*p* ≤ 0.001, effect size poor)/right (*p* ≤ 0.001, effect size poor) reduced. The maximum pressure and the left/right pressure increased with increasing BMI (*p* ≤ 0.001, effect size weak). An increase in pressure was also correlated with the body weight distribution parameters in the right (*p* ≤ 0.001, effect size moderate)/left (*p* ≤ 0.001, effect size weak) rearfoot. In the forefoot on the left, the pressure increased with increasing BMI (*p* ≤ 0.03, effect size poor).

### Age group comparisons

**Fig. 2:**
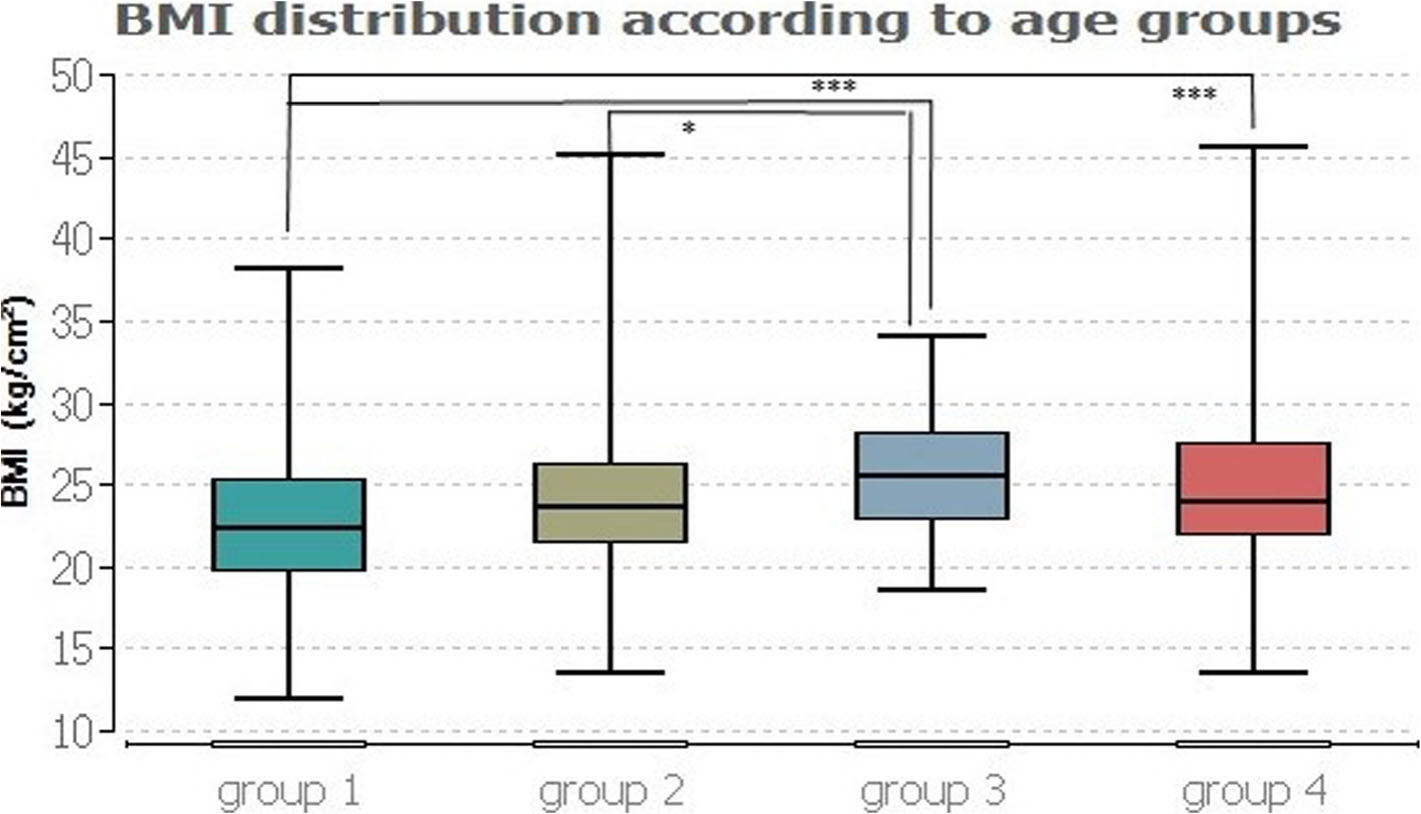
BMI distribution according to age groups

**Fig. 3:**
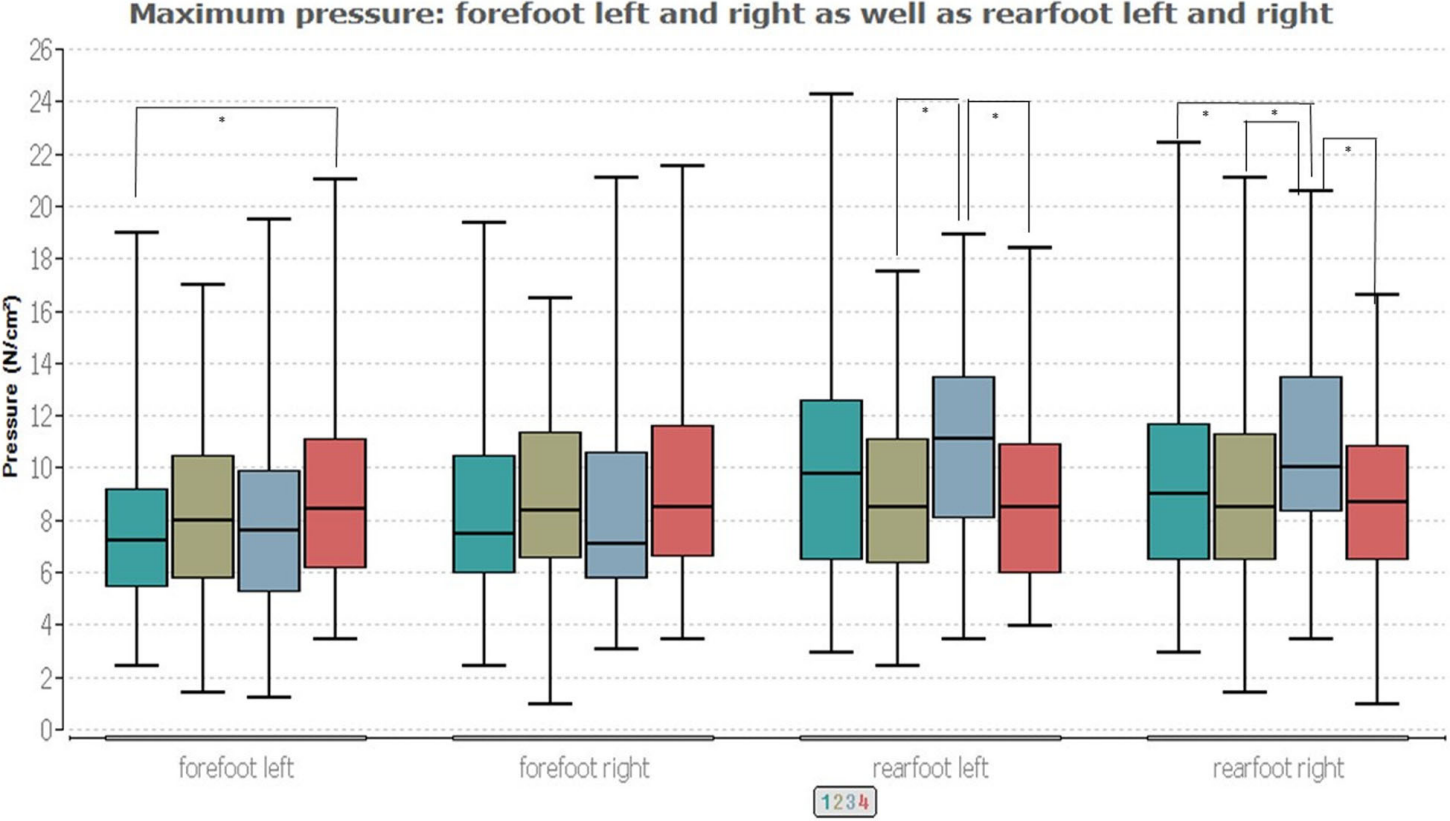
Distribution of the maximum pressure among groups and foot regions

Figures 2 and 3 contain the results of the age group comparisons.

Figure 2 illustrates the BMI distribution in relation to the four age groups. The applied Friedman test shows a chi-squared value of *p* ≤ 0.001. The subsequent post hoc test including the Bonferroni-Holm correction indicates significant group differences between groups 1 and 3 (*p* ≤ 0.001), 1 and 4 (*p* ≤ 0.001), and 2 and 3 (*p* ≤ 0.02). Accordingly, group 3 has the highest median BMI of 25.46 kg/cm^2^, while the BMIs of the other 3 groups range between 22.35 (group 1) and 23.93 (group 4).

There were no significant differences (*p* ≥ 0.05) between the age groups with regard to weight distribution. The maximum pressure in the left and right foot as well as in the right forefoot area does not show any age-related group differences (p ≥ 0.05).

Figure 3 illustrates group differences in the left fore- and rearfoot (*p* ≤ 0.03/0.001) and in the right rearfoot (*p* ≤ 0.01). In the left forefoot, a significance of *p* ≤ 0.02 is observed between the youngest (group 1, 7.22 N/cm^2^) and oldest (group 4, 7.6 N/cm^2^) age group. In the left rearfoot, there is a significance between groups 2 and 3 (*p* ≤ 0.01) and groups 3 and 4 (*p* ≤ 0.01). Groups 2 and 4 have the same median of 8.5 N/cm^2^, while that of group 3 is 11.1 N/cm^2^. In the right rearfoot, there are group differences between groups 2 and 3 (*p* ≤ 0.02), 1 and 3 (*p* ≤ 0.05), and 3 and 4 (*p* ≤ 0.01). Here the medians lie between 8.5 N/cm^2^ (group 2) and 10 N/cm^2^ (group 3).

### Gender comparison

The comparison of the two gender did not lead to any significant differences (*p* ≥ 0.05).

## Discussion

In the current study, 416 (208 m/208f) healthy volunteers aged 18 to 65 years were analyzed in order to establish standard reference values for the weight and the maximum pressure distribution and to correlate them with age and BMI. The women of the present study were of average normal weight (23.2 kg/m^2^) and non-obese, which reflects the average female German population [36, 56]. The men had an average BMI of 25.1 kg/m^2^, so they were considered as normal to pre-obese. This is also consistent with the German health study by Mensink et al. [36]. The detailed BMI distribution according to age groups (group 1, 18–30 years; group 2, 31–40 years; group 3, 41–50 years; group 4, 51–65 years) shows that the age groups 1, 2, and 4 are of normal weight (BMI 22.35–23.93 kg/cm^2^), while the age group 3 is classified marginal pre-obese with 25.46 kg/cm^2^. This could be due to an unequal gender distribution in favor of the men in this group, with the men in this study having a higher BMI on average. With rising socioeconomic status, the proportion of obesity in men and women decreases [35]. However, socioeconomic status was not investigated in the present study, and therefore, it cannot be assessed whether women have a higher socioeconomic status. Why the BMI is larger in the 41–50 years age group in particular cannot be explained in the context of this study. With regard to the BMI distribution in the respective age groups, a comparison with other German surveys [36] shows that similar comparative data are available here.

In terms of weight distribution, there is a totally balanced weight relationship (50.07%:50.12%) between left and right body side, while there is less load on the forefoot (left 45.49%, right 44.26%) and more load on the rearfoot (left 54.14%, right 55.09%).

The maximum pressure distribution is balanced (left side 11.05 N/cm^2^, right side 11.0 N/cm^2^), too. The median values of both forefeet (left 8.23 N/cm^2^, right 8.59 N/cm^2^) and rearfeet (left 9.60 N/cm^2^, right 9.51 N/cm^2^) are similar and indicating a higher pressure load on the rearfoot area compared to the forefoot area. This correlates with the data from the weight distribution. More weight on the rear foot also results in increased pressure.

According to Obens [12], the body weight distribution was 50%:50%; the body weight distribution between both feet is thus balanced. In the forefoot, there was less weight distribution (left 45.49%, right 44.26%) than in the rearfoot (left 54.14%, right 55.99%), from which, on closer inspection, an increased shift of the balance on the rearfoot is to be noted on the right. On average, more pressure is exerted on the rearfoot than on the forefoot [1, 13, 14, 57].

Additionally, for young, healthy men as well as for equivalent women, it could also be confirmed that there is an almost balanced body weight distribution between the left and right side of the body [13, 14]. Furthermore, the increased load is always on the rearfoot. However, when comparing the available results with those of other studies [1, 13, 14, 57], it must be taken into account that the present subjects have a less asymmetrical forefoot-rearfoot weight distribution. These studies referred to selected smaller age groups, whereas in the present study a very broad age spectrum was examined.

According to Scharnweber et al. [13], who examined only male participants, the most heavily loaded foot quadrant was the left rearfoot in contrast to Ohlendorf et al. [13], where female subjects have main load in the right rearfoot. Same results (main load right rearfoot, 34.3%) can be seen in the present study although both genders of different ages were measured. This discrepancy should be further investigated in the framework of future analyses. However, the present values for the left-right and forefoot-rearfoot weight distribution are very similar to the results of other studies [1, 12–14, 57].

With increasing BMI, the body weight shifts to the left and right rearfoot (*p* ≤ 0.001, poor effect size). Since this correlation is only very poor and, as shown in Fig. 1, the percentage range of change is very small, these results should only be considered as a trend and should be examined more thoroughly in further analyses. Thus, hypothesis 2 can be verified. Ohlendorf et al. could not detect any significant changes of weight distribution in truck drivers sorted by BMI groups according to the WHO classification [58] although they noted a rising BMI with an increasing number of working years. In the left foot, a weak correlation of weight transfer to the forefoot with increasing age was found, but this could not be confirmed after dividing all subjects into four age groups. Genthon et al. [59] describe that an imbalance between obesity and muscle strength is the cause of levels of instability. A rising BMI increases instability. A negative effect on postural control due to obesity was also demonstrated by Salsabili et al. [60] and Ku et al. [61]. They found that a higher BMI results in more fluctuation, less stance stability and less motor response. In contrast, the result of this study is that the forefoot is more heavily loaded as a result of these things. However, this was not examined in detail in the present study.

Age seems to have no influence on the balance distribution. Several authors have noted that as age increases, changes in postural control have an impact on balance. But Schwesig et al. [45] describe the greatest postural stability at the age of 20.1–30 years. After the age of 50 there is a decrease in performance. Mittermaier and Fialka-Moser [44] described that performance increases again at the age of 60.1–70 [45]. Hypothesis 3 that age has no influence on the balance distribution can thus be confirmed.

With regard to the maximum pressure distribution, a balanced distribution can be seen in the left-right comparison, just as with the weight distribution. Likewise, the pressure in the rearfoot is also higher than in the forefoot. Higher pressure values are achieved in the rearfoot, which agrees with the results of Birtane et al. [62] and Hills et al. [63]. Further, they have found that there is increased pressure in the rearfoot when the postural balance is disturbed by obesity which is in line with the present results: as BMI increases, so does the maximum pressure in all areas with a weak to moderate correlation [64, 65]. Fjeldstad et al. [66] describe obese stature as the cause of balance impairment. Park et al. [32] also confirmed that the pressure values of the heel and the big toe in the obese group increased in comparison with young normal weight subjects and those with increased BMI. An increase in the pressure on the big toe was not recorded in the present study, however, the results of the pressure increase of the rearfoot coincide with an increase in pressure at the heel. The existing studie s[62–65] often describe a comparison between the condition before or after an intervention. The results of independent studies should be better classified, so making it possible to yield a statement in which the tolerance ranges include the evaluation parameters.

In addition to the BMI, age also has an influence on the maximum pressure distribution. The varying changes in the age groups may be related to the BMI, especially between younger (18–40 years) and older (41–65 years) subjects. With increasing age, the maximum pressure in the left foot shifts from the rearfoot to the forefoot (*p* ≤ 0.02 and 0.03; poor effect size). In the right forefoot the pressure also increases with age (*p* ≤ 0.02; poor effect size). Since the effect strength is only poor here, these results should only be classified as a trend. However, no significance could be determined for the rearfoot on the right. This asymmetrical pressure distribution has not yet been the subject of other studies so far, so there is no comparative literature and should be further investigated. Lalande et al. [15] found in a recent study on standard values of pressure and foot areas measured in the static state that maximum and mean plantar pressure do not correlate with age but with weight, body mass index and shoe size. But plantar pressure distribution in static stance has been associated with pain and pathological profiles in older adults [67], as well as obesity status in children [68].

Furthermore, these standard values of the weight and maximum pressure distribution appear to be gender-independent. Pomarino et al. [40] and Lalande et al. [69] also found no influence of gender on the load distribution when considering adults. Therefore, hypotheses 1 and 4 can be verified.

In relation to the limitations of this work there are factors which have not been taken into account but which could possibly have an influence, such as hand or leg dominance.

The number of left-handers in this study was very low at 9.6%, in contrast to 90.4% of right-handers, but is in line with the European average (10–15%) [70]. In the European average, 91% have a dominance of the right hand with a simultaneous dominance in the right foot (83%). However, there are authors who have found that handiness does not always correspond to the load on the ipsilateral foot [71, 72]. These tests, which are used to check the foot, were not weight or pressure measurements. Due to the divergent findings in this regard, they are not considered in this analysis and should rather be investigated in a new study.

The question also arises whether the pressure plate is a suitable instrument for the analysis of standard values. In this context, Baldini et al. [73] found that the sensors offer good reliability and reproducibility. However, room temperature should be taken into account, as the sensors are temperature sensitive [74]. For this reason the measurements were always performed under constant conditions. In addition, the software should also be able to provide information about the exact localization of the pressure distribution in the foot in order to be able to compare this better with other studies. This could lead to more precise information about the localisation of pressure peaks and the development of metatarsalgia. Although the measured time of 5 s appears short, the software gives very accurate values. It takes 5 s x 120 measurements per measuring point. The used range is determined over all measurements, i.e. the largest/smallest X or Y position with a pressure value not equal to 0 is searched for. This range is divided into quadrants in the middle between the minimum/maximum X or Y positions (left/right/front/back). The value specified by the software is the average of all time steps.

Another limiting aspect is the short measuring sequence of 5 s. Since this measurement is part of a study in which weight and pressure distribution is recorded simultaneously with a three-dimensional back scan, the measurement duration of a measurement sequence of 5 s had to be taken, since recording the upper body posture with the back scanner takes just that time. Several measurements of habituation carried out in advance should reduce bias in this respect. In future studies, the limiting factors should be included in order to obtain more precise results or to confirm these results.

## Conclusion

The standard values for the weight and pressure distribution of healthy women and men aged 18–65 years correlate with the already available data of young, healthy subjects, i.e. men (18–35 years) and women (21–30 years), and can be considered representative while no gender difference could be detected. The weight distribution of the left and right foot of the subjects can be described as balanced. The rearfoot has 20% more load than the forefoot. In addition, a higher load on the right rearfoot compared to the left rearfoot could be determined. Similarly, the pressure values in the right and left foot are balanced. There is also a higher pressure load in the rearfoot than in the forefoot. There are significant differences in weight and maximum pressure distribution in the forefoot and rearfoot in the different age groups, especially between younger (18–40 years) and older (41–65 years) subjects. Age seems to have a greater influence than BMI on the values. In the future, these standard values can be used for analysis before, during and after therapy to obtain an objective evaluation of the treatment result.

## Data Availability

The datasets supporting the conclusions of this article are included within the article.

## Abbreviations

TR: Tolerance range
CI: Confidence interval
